# Modifying Influence of Malaria upon Diseases of the South and West

**Published:** 1849

**Authors:** Westel W. Sedgwick

**Affiliations:** Little Rock Illinois


### ARTICLE IX.

*Modifying Influence of Malaria upon diseases of the South and West.* By Westel W. Sedgwick M. D., of Little Rock Illinois.

Probably there are not many, if any, of the profession who have resided for any length of time in the valley of the Mississippi, who have not noticed the modifying influence which malarial poison has over most if not all of the diseases of the country, and yet, most medical men of this region though they may have noticed its influence to a certain degree, do not, as I believe, consider how much it should have to do in modifying the treatment. The reason for this is obvious. Our authors have either not understood this point themselves or, they have failed to give it that attention which its importance demands.

We require and should have a work to which we can refer for information upon this point, based upon western practice and experience. Eberle's Practce, which has perhaps had as good a reputation as any other work is entirely deficient in this particular. The same may be said, to a certain extent, of Watson's Practice and others.

The consequence is that physicians, especially such as have been educated in the East without the advantages of the teachings afforded by western schools on the diseases of our climate, must depend wholly upon their own judgement in each particular case, until by experience the true plan of treatment is learned and adopted. It mu at be admitted that this is a difficult task for them, when we consider the vast difference between the diseases of this climate, as compared with those of the Eastern states, the one requiring depleting, the other supporting treatment as a general rule.

My observation has led me to the conclusion that there is scarcely a disease in this climate that does not require soone or later, a tonic or supporting course of treatment, whether it be in winter, spring, summer, or autumn. Some physicians believe that it is only the diseases of summer and fall that require such a course of treatment; whilst in those of winter and spring, depletion should be resorted to. Will the success of this practice justify it? I answer no, but, this has been reccommended by Eberle and others who practiced where diseases were of a phlogistic character, hence the practice.

I have by no means found it necessary or even safe in my practice, but on the contrary prejudicial and dangerous for the safety of the patient, to follow this course of practice in this malarious region. I do not mean to say that I would abandon all other remedial agents for tonics. On the contrary I would make use of all other means indicated, never, however, losing sight of the modifying influence of malarial poison. By watching their effects closely I have become more and more convinced of the propriety of using quinine. wine, brandy, and carb. ammonia in many diseases in which an Eastern physician would consider such treatment detremental to the patient.

Some of the diseases to which I more particularly allude, are inflammation of the throat and tongue cynanche tonsillaris, pleuritis, peripneumonia, rheumatism, continued fevers, diarrhœa, cholera infantum, convulsions of infants, &c., in fact there is scarcely a disease in this climate in which, to use a familar expression, the "ague" has not somthing to do in producing the derangement. Perhaps some may question the propriety of giving quinine, wine, brandy and ammonia in inflammations, such as pneumonia. It is my opinion, however, that patients with what is called "lung fever" have for want of stimulants and tonics sunk under the prostrating influence of malarial poison.

It is necessary to use them sufficiently to support the patient, while at the same time other means may be resorted to combating inflamatory action. This I conceive to be the only true and rational course of treatment in this disease, for if we commence with and depend upon depletion alone we shall soon have a state of debility which even by the help of stimulants we shall not be able to remove. The comparative value of the two modes of treatment is shown in the following case,

On the 23d. of March last I was called in consultation to see a patient about 17 years of age; who had been laboring under pneumonia five days. The treatment up to this time, as I was informed, had consised in the adm inistration of purgatives, sedatives, alteratives, and anodynes. The attending physician had given up all hopes of the patients recovery.

I proceeded to examine the patient and found the features sunken and pale, lips blue, profuse perspiration over the face and chest, extremities cold, breathing irregular, hurried and laborious, the right lung almost wholly impervious to air, crepitent rattle distinct in some portions, but a total absence of the healthy vesicular murmur, left lung also partially diseased, tongue dark yellow or brown and dry, teeth covered with sordes, coughed some and with great difficulty succeeded in occasionally raising a frothy bloody matter; in fact impending suffocation threatened the patient's life. Pulse a mere undulating line. This was the condition of the patient at 10 o'clock A. M. Prescription. Quinine gr. 1, sulph. acid ggt. 1, vinum f ʒi, every half hour. Sub. mur. hydrarg. grs. iii, every four hours with a blister over the chest. Ten o'clock, P. M., pulse more full and distinct, constant low muttering delirium, jactitation, picking of the bed clothes. Prescribed quinine grs. iv, sub. mur. hyd. grs. iv, a small opiate to prevent action of the bowels, repeated every three hours alternately with carb. ammonia, grs. iv, camph. grs ii. The blister having drawn well, it was dressed with mercurial ointment. Saw him next morning---not much alteration. Continued the treatmeut---ordered for drink slippeɹy elm and toast water. Evening---less joctitation, body and lower extremities moist, tongue not so much parched, not much change in the condition of the lungs, bowels moved by enema, discharge dark and offensive ; continued the treatment, and appled another bliister over the right side of the chest. Next morning found him manifestly worse. By the directions of the attending physician, the camph. and am. had been omitted. Extremities cold, palor of the countenance, eye-ball fixed immovably. Gave him pretty freely of a solution carb. am., applied sinapisms to feet and legs, had him thouroughly rubbed with spirits and an infusion of red peper, and ordered the former treatment to be continued, and on no account to be discontinued. From that time forward, this patient continued to mend, and has since to all appearance, entirely recovered. I could cite numerous other cases illustrative of the efficiency of the treatment recommended above. In cases where I am called in the first instance I commence with the supporting treatment, and do not suffer my patients to sink so low. Usually the quinine and ammonia will be sufficient but often in the later stage I use brandy or wine. This course should be steadily persevered in.

